# Prerequisites for sustainable care improvement using the reflective team as a work model

**DOI:** 10.3402/qhw.v9.23934

**Published:** 2014-10-23

**Authors:** Lise-Lotte Jonasson, Gunilla Carlsson, Maria Nyström

**Affiliations:** Academy of Care, Working Life and Social Welfare, University of Boras, Boras, Sweden

**Keywords:** Reflective team, care improvement, sustainability, phenomenon, phenomenography, lifeworld

## Abstract

Several work models for care improvement have been developed in order to meet the requirement for evidence-based care. This study examines a work model for reflection, entitled the reflective team (RT). The main idea behind RTs is that caring skills exist among those who work closest to the patients. The team leader (RTL) encourages sustainable care improvement, rooted in research and proven experience, by using a lifeworld perspective to stimulate further reflection and a developmental process leading to research-based caring actions within the team. In order to maintain focus, it is important that the RTL has a clear idea of what sustainable care improvement means, and what the prerequisites are for such improvement. The aim of the present study is, therefore, to explore the prerequisites for improving sustainable care, seeking to answer how RTLs perceive these and use RTs for concrete planning. Nine RTLs were interviewed, and their statements were phenomenographically analysed. The analysis revealed three separate qualitative categories, which describe personal, interpersonal, and structural aspects of the prerequisites. In the discussion, these categories are compared with previous research on reflection, and the conclusion is reached that the optimal conditions for RTs to work, when focussed on sustainable care improvement, occur when the various aspects of the prerequisites are intertwined and become a natural part of the reflective work.

Swedish healthcare legislation supports the view that caring, in the same way as medical treatment, should be evidence based (i.e., based on research and proven experience) (SOSFS, 2011: 9). In order to meet this requirement, healthcare organizations, both in Sweden and elsewhere, have experimented with several work models for care improvement with different focusses, for example, system performances, organizational development, and team coaching (Wallin, Ewald, Wikblad, Scott-Findlay & Arnetz, 2006; Batalden & Davidoff, 2007; Godfrey, Andersson-Gare, Nelson, Nilsson, & Ahlström, 2013; Shaw, Howard, Etz, Hudson, & Crabtree, 2012).

However, work models for reflection are not always well received in actual practice. According to Hackman and Wageman (2005), many organizations focus on tasks and routine procedures, thereby avoiding discussing interpersonal relations. Moreover, when the tasks are completed, there is little or no time for joint reflection. Thus, some developmental possibilities in which a team can play a valuable role in organizational success are lost (Martin & Bale, 2007). The reverse is also true. If a team has the right mix of skills and practices for sharing work and providing for frequent and clear communication, the chances of developing practice increase (Katzenbach & Smith, 1993). Team members can also help each other to integrate new meaning and to understand more about their own competence, and teamwork can make it possible to affect other people's thinking, making this work as a starting point to further the development of practice (Berlin, 2010; Kozlowski & Ilgren, 2006; Oborn & Dawson, 2010).

It seems fair to presume that the benefits of teamwork are also relevant for the sustainable development of care. Based on a literature review, Meijers, Janssen, Cummings, Wallin, Estabrooks, and Halfens (2006) suggest that care improvement depends on the extent to which research is utilized, the role of the nurse, access to resources, the organizational climate, multifaceted support, and time available for research activities.

Yet there are some critical viewpoints, suggesting the difficulties in taking on different leadership roles in teams (Berlin, 2010). There are also on-going discussions on sustainable development itself. Nolin (2010) suggests six main ideas included in the concept of “sustainable development.” The patients’ quality of life, wellbeing, and participation appear to be the most relevant issues for sustainable care improvement. These issues are defined as social dimensions of sustainability, even if they focus on health and equal treatment.

Emphasizing these dimensions requires the caregivers and the team to reflect on what sustainable care improvement means from the patient's point of view. Our approach to meeting such requirements is a work model for reflection, called the “Reflective Team,” abbreviated to RT (Nyström, 2013). In an RT, reflection is a conscious act of careful thinking (Ekebergh, 2001) in which previous caring experiences are articulated, challenged, and placed in relation to caring science.

The concept of a “reflective team” has been used several times earlier. One of the researchers most widely mentioned in this connection is Andersen (1987), who introduced reflective teams in the field of psychotherapy in the late 1980s. His model emerged from the experience of working with families in counselling teams, and it allowed for an open, transparent process of reflection focussed on questioning (Pender & Stinchfield, 2012). Today, Andersen's model is used in several other contexts, for example in caring supervision (Morrison, 2009; Nordvang, 2009).

Our use of the concept of a “reflective team” does not follow the definition or practice of any other author, although it does share some of their ideas, such as non-judgemental and solution-focussed reflection. The idea behind the present RT model is that plans for sustainable care improvement can be developed from a competence that already exists but needs to be reflected on, articulated, and further developed in order for it to contribute to concrete measures. Similar ideas have recently been presented by Aitken (2011), suggesting that discussions about care must be incorporated into practice in order to provide for sustainable care improvement, with nurses discussing clinical evidence before implementing research results into daily programmes.

Our RT concept is not bound to a specific healthcare context and can be applied in different ways depending on the requirements and expectations that exist in various healthcare specialities. The main idea behind the model is that inarticulated caring skills exist among those who work closest to the patients. An RT comprises 5–10 such carers working in the same caring context, led by reflective team leaders (henceforth RTLs). Several RTs can take place in the same caring unit. Further development is stimulated when the team members’ perceptions of their own knowledge become clearer. If this process is successful, it seems reasonable to assume that it will increase the prospects for successful change. Thus, the goal is, through reflection, to highlight the RT members’ own caring experiences and link them to relevant research in order to create action plans for sustainable care improvement (i.e., care that uses research results in which the patient's perspective is the main consideration). RT can be limited to one profession or linked to several professionals who all work close to the patients. Both approaches have been tried, and other combinations are also possible (Nyström, 2013).

The theoretical foundation for this RT model is the lifeworld theory (Dahlberg, Dahlberg, & Nyström, 2008), that is, the world as it is apprehended and experienced by human beings. The theory of the lifeworld is a part of the phenomenology that was introduced by Edmund Husserl more than 100 years ago (Dahlberg et al., 2008). From a lifeworld perspective, it is not relevant to ask whether or not a phenomenon exists, but how it is experienced by human consciousness. From that perspective, all human beings have natural perceptions of their lives, meaning that they usually take them for granted without any active reflection. Hence, comprehensions concerning new caring situations derive from both conscious and unreflected experiences of similar situations. During RT sessions, the RTL encourages the team members to bring their experiences to the surface, and then further reflect on them.

RTs have so far been implemented and researched as part of a major project in collaboration with emergency care, psychiatric care, and neurological care (Carlsson, Hantilsson, & Nyström, 2014; Nyström, 2013). It has been found that to implement RTs successfully, certain obstacles must be considered in order to avoid participants getting caught up in uncertainty, using immediate care as an excuse to miss an RT session, and having a structure that is too planned or too loose. It is also important to prevent hierarchical patterns from dominating the sessions. The challenge is to abandon the easiest way out, move away from the most comfortable solutions, and participate in a dialogue that has the potential to open up one's mind to new ways of thinking.

The previous findings in the RT project led to the following research question: What are the prerequisites for sustainable care improvement using RT for concrete planning? The current study aims to explore this by using the concept of “sustainable care improvement” in the sense of including patient wellbeing and participation (Nolin, 2010).

## The clinical intervention

Caregivers from four different professions (registered nurses, staff nurses, mental care workers, and medical secretaries who receive patients in receptions), from three caring contexts in the western region of Sweden, were trained at the University of Borås to work as RTLs. During the training course, which took place once a month over 1 year, they implemented an RT in their own caring units. Together with researchers in caring science, who were responsible for the university course, they helped each other to identify research that could stimulate reflection and to solve the implementation problems that occurred.

The starting point for the reflective process was the team members’ professional experiences. The team members were caregivers drawn from different professions, including physicians, registered nurses, staff nurses, and medical secretaries. The RTLs stimulated their reflections concerning existing knowledge and skills, and placed these in relation to caring research. From this starting point, the intention was to plan caring actions.

## The study: phenomenographic research

The study, which is presented in the current article, is the third in the major project on RT which has been described above (Carlsson et al., 2014; Nyström, 2013). It builds on new interview data that focus on the RTLs’ perceptions of the prerequisites for sustainable care improvement *as lived experiences*. Thus, the epistemological perspective is the same as the theoretical foundation for the current RT model (i.e., the lifeworld perspective that requires phenomenological openness towards a phenomenon). As already mentioned, this perspective is not directly interested in whether or not a phenomenon exists, but how it is experienced. The research attitude is therefore characterized by openness and flexibility towards different understandings of the research phenomenon, not decisions or definitions of concepts, which are determined before the analytic phase.

In this study, attention is directed to variation in the investigated phenomenon of “prerequisites for sustainable care improvement using RT for concrete planning.” Therefore, phenomenography was chosen as a suitable research method.

Phenomenography is congruent with the phenomenological lifeworld perspective and investigates how people think about the world. It aims to discover the qualitatively different ways in which people experience, conceptualize, realize, and understand various aspects of a phenomenon, based on lived experiences of it (Marton, 1986). This means that neither the phenomenon per se nor the participants as subjects are of interest in the empirical analysis.

### Data collection

Data were collected by means of interviews with nine RTLs with professional backgrounds as registered nurses (six) and staff nurses (three). All were women. The interviews were semistructured and served to elicit the lived experiences of the “prerequisites for sustainable care improvement using RT for concrete planning.” The interviewees were invited to reflect on the significance of the following issues:

- collaborative climate
- evidence-based care
- patient perspective
- plans for care improvement

In order to stimulate reflections on the research phenomenon, follow-up questions were posed such as: Can you explain this further? What was it like for you? Can you give an example? Each interview lasted about 1 h. They were audiotaped and transcribed verbatim. The transcription of each interview resulted in 8–11 pages of text. The whole data set contains 90 pages of single-spaced text.

The transcribed interviews were analysed in accordance with the ideas of phenomenography. This qualitative research method was developed 30 years ago, primarily for use in educational research, as a method for identifying and systematizing forms of thought in order to describe aspects of reality (Marton, 1981). Following the lifeworld perspective, it is not the interviewees who are in focus but the research phenomenon as it appears in the data and its variations. Phenomenography builds on the assumption that things or events can be experienced in qualitatively different ways. A number of different understandings are identified, categorized, and described as a qualitative variation in understanding concerning the investigated phenomenon. This distinction, between what something is and how it is conceived to be, is in line with the lifeworld perspective, and it is also an essential aspect of phenomenography. The focus on how a phenomenon is perceived is called the second-order perspective, and this perspective is used in phenomenography (Wenestam, 2000).

### Analyse

The first step in a phenomenographic analysis is familiarization, which means that the researchers familiarize themselves with the material by carefully reading the manuscripts. The second step is compiling statements from interviewees in answer to a certain question, which makes it possible to identify the most significant elements in the data. The third step is the condensing of individual statements in order to identify the central parts, and the fourth is a preliminary classification and grouping of similar statements. The fifth step is a preliminary comparison of categories, and the sixth is naming the categories. The last step is making a contrastive comparison of categories, which includes a description of the character of each category (Sjöström & Dahlgren, 2002).

All of the authors were equally involved in the analysis. The transcribed interviews were read in their entirety, and attention was then directed towards similarities and differences in the statements. Different characteristics of understanding were identified by all of the authors, and the emerging pattern of qualitatively different categories was discussed in the research team. Once the categories were described in writing, the authors took responsibility for one category each with regard to describing the variations in understanding and conceptualization. The written category descriptions of understanding were then discussed once again in the research team, and quotations from the different interviews that illuminate variations in the whole data set were chosen for the article.

Thus, in the findings below, the categories are described and illustrated with fairly simple characteristics that are basically made up of the RTLs’ ways of reasoning about their own understanding. It should be noted, however, that one interviewee's composite understanding can be represented in two, or even three, categories. This is in line with the idea that it is the phenomenon which is of interest, not the subject making the statements. It also connects with the fact that one person can have different understandings of the same phenomenon. This aspect of phenomenography is called decontextualization (Friberg, Öhlén, Nyström, & Dahlberg, 2000).

### Ethical considerations

Information about the study was given to the manager of the clinic, the director of the department, and the personnel manager; all of whom approved the study. Informed consent, both written and verbal, was obtained from all participants. Both Helsinki Declaration (World Medical Association Declaration of Helsinki, 2014) and standard procedures were followed regarding informed consent and confidentiality (SFS, 2003: 460). The whole RT project is approved by the Ethics Committee of the Medical Faculty at the University of Gothenburg, Dnr 596-09.

## Findings

The conditions for care improvement are of an individual, interpersonal, and structural nature. These conditions are probably intertwined, but, according to the phenomenographic principle of decontextualization, they can also be divided into the three different categories of prerequisites which are presented in Table I.

**Table I:** Summary of categories.

Categories of prerequisites	Subcategories
A reflective attitude	Reflective thinking
	Reflective listening
	Reflective doing
	Reflective professional developments
Mutual interpersonal involvement	Interpersonally shared goals
	Interpersonally shared knowledge
	Permissive interpersonal climate
	Joint interpersonal reflection
Structures for sustainable care improvement	Structures for collaboration
	Structures for refinement of professional skills
	Structures for application of new methods

## A reflective attitude

The first prerequisite for sustainable care improvement is an individual reflective attitude, with a willingness to think, problematize, and think again and, by extension, listen in an open and active way. A reflective attitude, although a cornerstone of personal professional development, is nevertheless also directed to colleagues in various ways. The reflective attitude is clarified below as reflective thinking, reflective listening, reflective doing, and reflective professional development.

### Reflective thinking

Reflective thinking has both a “before” and “after” dimension in relation to a caring situation that provides substance for reflective thinking, as well as ideas about one's own thinking. Such a praxis-oriented approach develops into a more general reflective thinking, focussing on how knowledge can be generalized and thus its use made possible in other situations as well. Within that process, the individual caregiver can also feel a desire to acquire more knowledge in order to further deepen their own reflective thinking.

I've certainly thought about it before (when something happens) but why did this happen today? So, I can imagine, I think in a deeper way now [after implementation of RT] than I did before.

It's interesting this with how we think about people. What is it that makes you think in the way you do? Going deeper into my thoughts and into my behavior patterns makes it clear that the questioning of old routines and care improvement are linked.Thinking about a specific caring situation can promote care improvement. This can in turn start chain reactions, and then it becomes extremely important. I believe that's why I have developed an interest in reflection.

### Reflective listening

Reflective listening involves active listening, which occurs best as a simultaneous process in which listening and thinking are intertwined. Professional listening is stimulated by encounters with patients, even if the time available permits only short meetings. This competence is experienced as requiring a capacity for empathy. Reflective listening also includes listening to colleagues. It is especially important to affirm different professional experiences as starting points for further reflections on how to handle caring situations.

When you listen properly you are present, meaning that “I have time for you right now.” Sometimes, people talk without being there. It is terrible when a person looks at something else or listens to something else when you are talking.

Caring is very much about being empathetic, but you have to really listen in order to understand the patient's perspective.

One must be sensitive to opinions from colleagues. At our caring unit, there are many opinions; therefore, it's important to allow for differences.

### Reflective doing

Reflective doing is preceded by both thinking and listening. It also includes opportunities to stop and evaluate what you are doing while you are still doing it. Reflective doing is further stimulated by colleagues’ questions, which create awareness of why one does something in a particular way. However, to do in a reflective way is not just to *achieve* something yourself. Reflective doing can also be to confirm colleagues that they have done something good, and this constitutes an attitude that shows itself in action.

Then I stop and reflect: “how do I do things?” or “can I do it in another way?” One can do the same thing in different ways depending on who you are, how you do things, and how you say things.

I can see in my own practice what is happening and how I can change that which was not so good “on the floor” so to speak. Thus there is always a care development inside me.

### Reflective professional development

A reflective attitude is closely connected to personal professional development. Developmental processes can start on a variety of occasions, for example during a practical case or an academic course. Openness to new knowledge is significant for development. The process can start when problems are acknowledged, for example that you are scared because you cannot handle a problem. Most important for reflective professional development is the willingness to learn. Learning is not only about training and/or gaining factual knowledge, but also about reflecting on a new basis and, thanks to that, becoming able to reflect further. Reflective learning can also give rise to a positive attitude to caring research and to personal professional development.

There was an incident in the emergency room that made us contact the psychiatric clinic. We found that our fear was rooted in ignorance or fear of this category of patients.

When you read one caring science article that touches you. You start to reflect on what you have read, and that can start a process for care improvement.

Then, I think that you just think of yourself. There are no care improvements without development of the self.

## Interpersonal mutual involvement

The second prerequisite for sustainable care improvement is interpersonal mutual involvement, which shows itself as a sense of togetherness during RT sessions. Encounters involving mutual respect make it easier to set common goals that are congruent with professional values and therefore easy to understand and share. Such encounters require a firm starting point with a permissive open attitude, in which old routines are questioned and new challenging questions are allowed. This is characterized as interpersonally shared goals and knowledge, a permissive interpersonal climate, and joint interpersonal reflection.

### Interpersonally shared goals

General caring goals are often decided at a higher level in the organization; therefore, they must be worked through and made interpersonally understandable. Understandability and manageability emerge when an RT session makes the goals a common concern for all involved. The sharing of interpersonally recognized goals can, for example, be expressed in various documents, such as guidelines and action plans. They are important and need an articulated and clearly expressed idea, with all carers moving in the same direction.

Care improvement is associated with common goals. The goals are set by the hospital management and we work with the implementation of the goals in every clinic. This requires reflection.

It [care improvement] is not about individual preferences but a joint commitment.

### Interpersonally shared knowledge

Through joint reflection personal, proven experience can be transformed into shared knowledge. Yet, such a development presupposes creative ways of sharing. In RT, this is achieved when individual proven experience is reflected on and transformed into common knowledge. This kind of sharing becomes especially clear when different professions participate in a reflective process connected to caring research. On such a basis, the importance of research becomes obvious. New opportunities for care improvement also emerge when team members ask each other questions, and start to question that which has so far been taken for granted.

Care improvement means sharing experiences after many years of caring. This takes place during reflection.

When I have been reading a research article, care improvement emerges when I communicate the findings to my colleagues. This is a starting point for further reflection.

### Permissive interpersonal climate

An atmosphere of sharing requires a permissive environment, characterized by openness and positive feelings of cooperation, with generosity and encouragement to open up and talk about thoughts and feelings. When a permissive climate exists, the reflection deepens, and plans for the improvement of care can be further developed. Caregivers introduce urgent issues that are perceived as important, and the ensuing reflections that arise make it easier to form concrete plans. Such a working environment creates conditions for community at work, which in turn forms the basis for professional relationships and closeness.

Hence, a permissive environment allows carers to challenge generally accepted norms and ideas. They begin to discuss: How are we doing that? Can it be done any better? Care improvement often benefits from questioning established routines and critical awareness of the risk of change for its own sake, even if the old routines work well. In an open and permissive environment, questions are constantly asked, and there are fewer unreflective thoughts that automatically fall back on old routines.

“A positive climate of cooperation is a condition for care improvement. An open working climate brings us close to each other.”

“Sharing in RT means that team members talk about what they believe is important. A majority of us open up and talk about it. If there is a positive climate of cooperation, the quality of care will also improve.”

“Care improvement means asking questions and challenging that which is taken for granted.”

### Joint interpersonal reflection

Joint reflection is of the utmost importance for interpersonal mutual involvement. That process starts when a routine or care situation needs improvement, and when all those involved are motivated to work together in a way that increases both cooperation and job satisfaction. Joint interpersonal responsibility makes everyone feel involved in giving extra attention to patients, who in turn feel secure in having staff who cooperate in their care. For care improvement to take place, it is important that all members of an RT participate in the sessions. The idea that they all have something to contribute is important. If knowledge is lacking in one area, joint reflection increases the chances of finding solutions.

Working in teams where everyone participates creates job satisfaction. When knowledge is lacking, we reflect together and discuss what to do for care improvement. Everyone in the RT is heard; everyone has something to share.

I believe that many patients have noticed that their carers’ work is characterized by community, not only in the RT but in the whole staff group.

## Structures that provide for sustainable care improvement

Structures for care improvement assume that learning and caring science research stimulate the implementation of new caring practices. This is further clarified as encouraging collaboration, acquiring knowledge, refining professional skills, and applying new methods.

### Structures for encouraging collaboration

All personnel in a care organization are dependent on each other for promoting care improvement. A structure that encourages cooperation provides sufficient resources for carers to exchange knowledge and ideas. The success of RT presupposes that the management specifies a time and place for different caring professions to reflect together in order to plan effective actions in accordance with specific needs. Thus, the time allocated for collaboration must be organized so that it is not the first thing to be dropped when the workload increases.

Management for care improvement also stimulates the exceeding of professional limits. Under such circumstances, RT becomes goal oriented, and everyone is expected to adhere to working methods which have been developed together.

I think it has been better [since we started with RT] because the more you cooperate, the more you get to know each other. I think that our striving for common goals is great for the patients.

### Structures for refining professional skills

A structure that encourages the acquisition of new knowledge stimulates an interest in it. This can show itself as a positive attitude towards academic support by, for example, encouraging interaction with people outside of the care organization, such as researchers in caring science.

I think we should have a contact person for collaboration with the university in all our clinics.

It is important to be open and receptive to the new, and that there is a science about caring, I think this is fascinating.

In their daily work, different professionals operate in different ways. During RT sessions, it is possible to recognize each other's areas as complementary to one's own competence. Thus, comparisons can make one's own expertise especially clear, in light of other professions’ skills and responsibilities. This, in turn, stimulates further reflections. Thoughtful methods and models that are evidence of such considerations are relevant to both practical and theoretical knowledge.

A structure which stimulates such refinement of professional skills puts trust in proven experience and different professional perspectives, allowing them to influence concrete planning and the reviewing of old routines.

We have to follow up with development. Methods are progressing. Sustainable care improvement requires us to use our available knowledge.

Challenging of old routines and care improvement are linked. But there are also good old routines, and it is important that we reflect together in order to arrive at what works well.

Developments mean we cannot carry on with the old routines. We have to follow the methods, moving forward to research and care development. We cannot stay with our old routines, that's not possible.

### Structures for applying new methods

New plans, which have been tested successfully, proceed in the RT with organized reflection on how to implement them in practical caring work. When the organizational goals are operationalized in concrete plans, the management becomes extremely important for the implementation phase. This requires cooperation at various levels in the organization and in different groupings. When an RT has highlighted an issue which requires concrete planning for the implementation phase, it is often important to include the whole caring unit in the further process.

That which we highlighted in our RT we took with us to the next staff meeting so that the further process could continue in the whole caring unit.

Adequate caring guidelines often focus on patients. When specifically expressed, such documents provide structure and stability. A management that keeps the unit together and organizes care so that planning, training, and implementation are included in a natural way in the daily care of patients is important for this kind of development. Thus, RT as part of an organizational structure can stimulate professionals to use their time in an effective way.

Writing action plans together gives structure to our caring work.

## Discussion

The method chosen for this study, phenomenography, assumes that forms of thoughts can be described in qualitatively different ways. Consequently, the main findings are presented in three separate descriptive categories, in which the prerequisites for sustainable care improvement were found to be of an individual, interpersonal, as well as structural nature. The individual level is closely connected to personal professionalism, the interpersonal to collective competence, and the structural to the overall opportunities afforded in a workplace. The phenomenographic idea of qualitative distinction, however, became problematic in the subcategories, which are not mutually exclusive. This is especially obvious in the first category, where reflective thinking, listening, and doing presuppose each other. Yet even if they usually occur simultaneously, it was possible in the analysis to distinguish them as separate elements, and we believe that it is precisely this possibility which is the advantage of the phenomenographic approach.

Another possible disadvantage is the circumstance that all of the interviewees were women. It is common in all phenomenon-oriented research to strive for both male and female informants. But it is also important that they have rather extensive experiences of the research phenomenon. In this case, it seemed reasonable to assume that the research question did not include a gender perspective. Therefore, RTLs were chosen, even if no males participated in the RTL education programme which served as a base for the selection of interviewees.

Research on similar issues has highlighted other aspects and has consequently been carried out using other research methods. When Shaw et al. (2012) investigated the process of reflection in a project on how team-based reflection affects quality improvement in a caring context, they found that physicians quite often dominated the reflective process. The physicians in their study were greatly instrumental in helping the team to reflect, making the staff critical of their autocratic decision-making style. In our RT project, similar issues have been highlighted in a previous study (Carlsson et al., 2014), where it was concluded that an RTL must prevent hierarchical patterns from dominating the RT session. In the present study, the same phenomenon is particularly obvious in the second category, which focusses on interpersonal mutual involvement. One tool that is available to the RTL, when it comes to creating conditions for interpersonally shared goals and knowledge in a permissive atmosphere, is the lifeworld perspective (Dahlberg, Dahlberg, & Nyström, 2008). As mentioned in this article, this is the theoretical foundation for our RT concept. A lifeworld perspective renounces right and wrong thinking by avoiding discussion and argumentation in favour of thorough reflection. Such a reflective process highlights one's proven experience, which is equally as important for evidence-based care as is research. In order to avoid dominance by one professional group in a multiprofessional team, Oborn and Dawson (2010) add the importance of translating knowledge across occupational boundaries. Otherwise, they conclude, the creation of a multidisciplinary structure may only support existing power hierarchies.

Our findings, at least partly, also confirm those of several other studies. Stroebel, McDaniel, Crabtree, Miller, Nutting, and Stange (2005) found, for example, that team-based reflection can enable a change which affects the organization and helps professionals to make sustainable improvements, if the process includes an understanding of the visions and learning reflection in practice. Such an atmosphere can also form the basis for trust with the reduction of social and relational boundaries, and increase the possibilities for effective change and care improvement.

In the short term, however, it is important to be aware that creative change requires time, and that time costs. Such important issues have previously been addressed by Stroebel et al. (2005), Shaw et al. (2012), and Carlsson et al. (2014). The third study points to the importance of recognizing the significance of indirect care, such as further training, supervision, and organized reflection, in order to improve care in a sustainable manner (Ekebergh, 2009). In the present study, this aspect is particularly obvious in the third category, which highlights structures that provide for sustainable care improvement, for example through academic support and time for the refinement of professional skills. This relation, between sustainability and organizational structures that encourage collaboration and goal-oriented healthcare, has also been found in a study by Wallin, Boström, Wikblad, and Ewald (2003). They place sustainability in relation to supportive leadership, use of human resources, knowledge of new research, and a willingness to implement research findings in clinical practice.

A creative leadership has been found to be extremely important in many studies, and we want to add that such leadership must encourage both individual reflection and joint reflection in this case between as well as during RT sessions (Nelson et al., 2011). Such workplaces encourage the staff to spare no efforts in order to accomplish evidence-based care. This is indeed important because several authors report a low utilization of research and a lack of the communication channels that are necessary to increase the application of research (Boström, Kajermo, Nordström, & Wallin, 2009). Moreover, an organizational climate which stimulates collaboration has been found to be very important for care improvement (Meijers et al., 2006). The findings in our study are in line with this and allow the presumption to be made that collaboration with reflection increases the ambition to implement new ideas and improve care in a sustainable way.

The issue of leadership is also closely connected to a leader's professional style. A supportive leadership in RT, as well as in other work models for care improvement, has been found to be extremely significant (Batalden & Davidoff, 2007; Carlsson et al., 2014). We believe that supportive leadership with a positive attitude towards staff, together with a reflective attitude, as in our first category, is comparable with authentic leadership (Dellve & Wikström, 2009; Wong & Giallonardo, 2013), that is, a leadership that is transformative, caring, and serving, supporting and encouraging individuals as well as team development. This in turn implies a leadership that can handle, in a trusting way, the dynamics related to professional groups and operational issues, and dealing with more strategic directions and management (Andersson, Åhgren, Bihari Axelsson, Eriksson, & Axelsson, 2011). Such leadership facilitates care improvement and makes the enhancements sustainable. In addition, it is also important to stimulate the achievement of improved patient outcomes, better procedures, and better learning (Batalden & Davidoff, 2007; Hughes, 2008).

## Conclusions

A work model for sustainable care improvement is enhanced by a professional approach in which attitudes, opinions, and discussions are further developed into creative reflection. This requires not only a personal reflective attitude but also a collegial environment, interested in mutual support in more thorough reflection. Optimal conditions for such development occur when there is an organizational structure at the caring unit which makes it possible to intertwine these factors so that they become a natural part of the work climate.
